# Clinical Characteristics of 162 Patients with Drug-Induced Liver and/or Kidney Injury

**DOI:** 10.1155/2020/3930921

**Published:** 2020-01-19

**Authors:** Xiaolin Wang, Xiangmei Chen

**Affiliations:** ^1^Medical School of Chinese PLA, Beijing, China; ^2^Department of Gastroenterology, Chinese Rocket Force Characteristic Medical Center, Beijing, China; ^3^Department of Nephrology, Chinese PLA General Hospital, Chinese PLA Institute of Nephrology, State Key Laboratory of Kidney Diseases, National Clinical Research Center for Kidney Diseases, Beijing Key Laboratory of Kidney Diseases, Beijing, China

## Abstract

**Context:**

Drug-induced liver and kidney injuries are the most common adverse drug reactions in the clinic, and they have similar pathogeneses.

**Aims:**

To analyze the clinical characteristics of patients with drug-induced liver and/or kidney injury.

**Settings and Design:**

This was a retrospective study.

**Methods and Materials:**

We analyzed data from 162 patients with drug-induced liver and/or kidney injury from 2008 to 2018 at the Chinese Rocket Force Characteristic Medical Center. Univariate and multivariate logistic analyses were performed on the drugs used, sex, age, weight, complications, and laboratory test results. Statistical analysis was performed using SPSS 25.0 statistical software.

**Results:**

(1) The most common drugs causing organ injury in this study were antineoplastic drugs, antibiotics, traditional Chinese medicine, lipid-lowering drugs, and nonsteroidal anti-inflammatory drugs. (2) Among 22 patients with drug-induced liver and kidney injuries, 68.18% had a hepatocellular pattern, 13.64% had a mixed pattern, and 18.18% had a cholestatic pattern. Among the three groups, the *P* value for creatinine was 0.002. (3) The *P* value for urinary protein between the isolated kidney injury group and the liver and kidney injury group was 0.028. (4) Multivariate analysis showed that, among the drug-induced renal injury patients and all injury patients, those with a higher neutrophil percentage had a lower risk of liver injury (OR = 0.574, 95% CI: 0.390–0.846; OR = 0.545, 95% CI: 0.396–0.749).

**Conclusions:**

(1) The serum creatinine level was higher in liver injury patients with the cholestatic pattern than in those with the hepatocellular or mixed pattern. (2) There was a significant difference in urinary protein between the isolated kidney and the liver and kidney injury groups. (3) Among patients with drug-induced organ injury, those with a higher neutrophils percentage had a lower risk of liver injury.

## 1. Introduction

The human liver is closely related to the kidney. In traditional Chinese medicine, there is the saying that the “liver and kidney have the same origin.” In Western medicine, there is the concept of a “hepatorenal disorder (HRD)” [1]. The liver and kidney are important organs for drug metabolism and excretion. In recent years, the problem of clinical drug-induced liver and kidney injuries has become more prominent.

We studied the data of 162 patients with acute drug-induced liver and/or kidney injury to determine their drug use, the differences in liver and kidney injuries caused by the same drug, the characteristics of liver and kidney injuries caused by different drugs, and risk factors for liver and kidney injuries. Our study may provide guidelines for rational drug use in the clinic.

## 2. Subjects and Methods

### 2.1. Research Subjects

From 2008 to 2018, patients with acute drug-induced liver and/or kidney injury were hospitalized at the Chinese Rocket Force Characteristic Medical Center.

Patients with drug-induced liver injury were diagnosed according to the RUCAM scale. The inclusion criteria for the research subjects were as follows: (1) drug exposure history within 3 months; (2) clinical chemistry parameters [2, 3] that fit any one of the following criteria: “more than or equal to fivefold elevation above the upper limit of normal (ULN) for alanine aminotransferase (ALT), more than or equal to twofold elevation above the ULN for alkaline phosphatase (ALP), or more than or equal to threefold elevation in ALT concentration and simultaneous elevation of bilirubin concentration exceeding 2× ULN”; and (3) liver function improved rapidly after withdrawal. The exclusion criteria were as follows: (1) liver injury caused by other diseases; (2) primary hepatobiliary diseases; (3) serious infections, shock, pulmonary insufficiency, heart failure, and other diseases that can cause hypoxic injury of whole body tissues; (4) age < 18 years; and (5) incomplete medical records.

The diagnosis of drug-induced kidney injury was based on the 2016 clinical guidelines in Japan [4]. The inclusion criteria for this study were as follows: (1) patients with acute kidney injury (AKI) with a drug exposure history within 3 months, (2) an abnormal estimated glomerular filtration rate (eGFR) based on serum creatinine using the Modification of Diet in Renal Disease (MDRD) formula developed by the simplified American Kidney Disease Diet Improvement Research Group:

MDRD formula: eGFR[ml·min^−1^·(1.73 m^2^)^−1^] = 186 × [Scr(mg/dl)]^−1.154^ × [age(year)]^−0.203^ × gender (male = 1.000, female = 0.742), after medication eGFR < 90 ml·min^−1^·(1.73 m^2^)^−1^or (3) the clinical manifestations of renal dysfunction such as hematuria or proteinuria under microscopy. The exclusion criteria were as follows: (1) acute renal injury caused by other diseases such as decreased blood volume or postrenal obstruction; (2) primary kidney disease; (3) severe infections, shock, pulmonary insufficiency, heart failure, and other diseases that can cause hypoxic injury to whole body tissues; (4) age < 18 years; and (5) incomplete medical records.

### 2.2. Research Contents

1. We checked the patients' detailed medical records and recorded data about age, sex, weight, and the medical history of the use of suspected drugs such as the drug name, dosage, administration method, course of treatment, and time from exposure to the onset. We also recorded data on patients' past histories including complications such as coronary heart disease, diabetes, hypertension, and other basic diseases.
2. Laboratory tests were performed on blood and urine samples. We recorded the results for the following items: alanine aminotransferase, glutamic oxaloacetic aminotransferase, alkaline phosphatase, total bilirubin, cholesterol, triglyceride, fasting blood glucose, creatinine, urea nitrogen, uric acid, C-reactive protein, white blood cell count, the percentage of neutrophils, and the international standardized ratio. We recorded the urine specimen test results including urinary protein and microscopic hematuria of the first morning urine. All laboratory inspection items were completed by the Laboratory Department of Rocket Force Characteristic Medical Center.

### 2.3. Statistical Methods

Our statistical analyses were performed with SPSS (Statistical Product and Service Solutions) 25.0 by International Business Machines Corporation:

1. The measurement data were analyzed by the Kolmogorov–Smirnov (K-S) test for normality. Normally distributed data are expressed as the means ± standard deviations. Student's *t* test (*T* test) was used for comparisons between two groups, and analysis of variance was used for comparisons among three groups. The nonnormally distributed data are expressed as the medians and interquartile ranges. The Mann-Whitney *U* test (*U* test) was used for comparisons between two groups, and the rank-sum test was used for comparisons among three groups. The count data are expressed as percentages. The comparisons between two groups were performed with the chi square test. *P* < 0.05 indicated statistical significance.
2. The possible risk factors were analyzed by binary logistic regression analysis. Forward: the LR method was used for the analysis. The entry standard was 0.05, and the deletion criterion was 0.10. *P* < 0.05 indicated a correlation.

## 3. Results

### 3.1. General Information

Data from 162 patients were collected, with more than 90% integrity of the medical records. There were 68 males and 94 females. The average age was 50.93 years, the youngest patient was 19-year-old, and the oldest patient was 85-year-old. There were 105 cases of drug-induced kidney injury, 25 cases of drug-induced liver injury, and 32 cases of liver and kidney injuries.

### 3.2. Results of Drug Analysis

**Figure 1:**
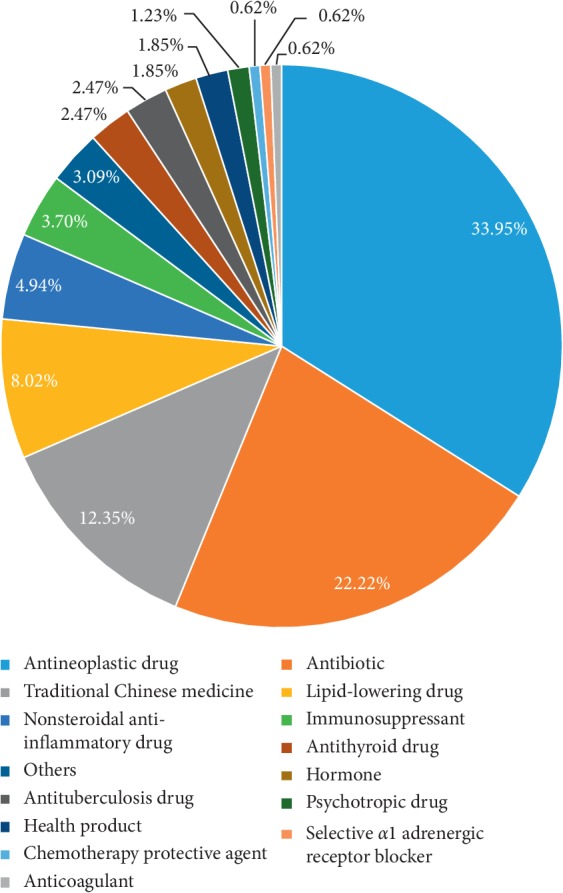
Drugs and the incidence of liver and/or kidney injury.

**Figure 2:**
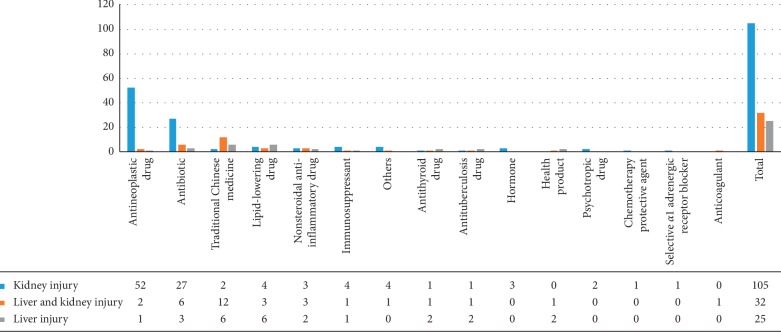
Different drugs causing liver and/or kidney injury.

1. In this study, the most common drugs causing acute liver and/or kidney injury were antineoplastic drugs (55 cases, 33.95%), antibiotics (36 cases, 22.22%), traditional Chinese medicines (20 cases, 12.35%), lipid-lowering drugs (13 cases, 8.02%), and nonsteroidal anti-inflammatory drugs (8 cases, 4.94%), as shown in Figure 1. In addition, immunosuppressants, antithyroid drugs, antituberculosis drugs, hormones, health products, sedatives, and psychotropic drugs also caused liver and kidney injuries. This is in line with Chinese drug use habits. Antibiotic abuse is common in China. Environmental pollution and food contamination are severe, causing the incidence of cancer to increase greatly. With the development in medical examinations, the early detection rate of cancer is increasing, and the use of antineoplastic drugs is increasing. In recent years, unreasonable abuse of traditional Chinese medicines and Chinese patent medicines has become common.
2. Among 55 cases of injury caused by antineoplastic drugs, 54 cases (98.18%) had kidney injury or simultaneous liver and kidney injuries. Among 36 cases of antibiotic-induced injury, 33 cases (91.67%) had kidney injury or simultaneous liver and kidney injuries. Among the 20 cases of injury caused by traditional Chinese medicines, 18 cases (90%) had liver injury or simultaneous liver and kidney injuries. Among 13 cases of injury caused by lipid-lowering drugs, 9 cases (69.23%) had liver injury or simultaneous liver and kidney injuries. Among the 8 cases of injury caused by nonsteroidal anti-inflammatory drugs, 6 cases (75%) had kidney injury or simultaneous liver and kidney injuries. These results are shown in Figure 2. In this study, antitumor drugs, antibiotics, and nonsteroidal anti-inflammatory drugs were found to be more likely to lead to renal injury, while traditional Chinese medicines and lipid-lowering drugs were found to be more likely to lead to liver injury.
3. In this study, the most common drugs causing acute liver injury were Chinese medicines, lipid-lowering drugs, and antibiotics, while the drugs causing acute kidney injury were antineoplastic drugs, antibiotics, and traditional Chinese medicines. These results are shown in Figure 2.

(2) Antineoplastic drugs and antibiotics were the two classes of drugs that caused the most acute drug-induced injury in this study. We divided patients into an antineoplastic drug group and an antibiotic group. The levels of alanine aminotransferase and total bilirubin were compared between the two groups to determine whether there were any differences in liver injury caused by these two types of drugs. The levels of creatinine, uric acid, and urea nitrogen were compared between the two groups to determine whether there were any differences in renal injury caused by these two types of drugs. The results showed that there was no significant difference in the effects of antineoplastic drugs and antibiotics on liver and renal biochemical tests, as shown in Table 1.
(3) Patients with antibiotic-induced kidney injury were divided into an isolated kidney injury group and a simultaneous liver and kidney injury group. We compared the serum creatinine, uric acid, and urea nitrogen levels between the two groups with *T* tests and compared the urinary protein levels and microscopic hematuria with chi square tests. There were no significant differences in renal function between the two groups, as shown in Table 2.
  The patients with liver injury caused by antibiotics were divided into an isolated liver injury group and a liver and kidney injury group. The levels of alanine aminotransferase, glutamyltransferase, alkaline phosphatase, and total bilirubin were compared between the two groups with *T* tests or Mann–Whitney *U* tests. There were no significant differences between the two groups, as shown in Table 3.
(4) Analysis of acute drug-induced liver injury combined with renal injury.

**Figure 3:**
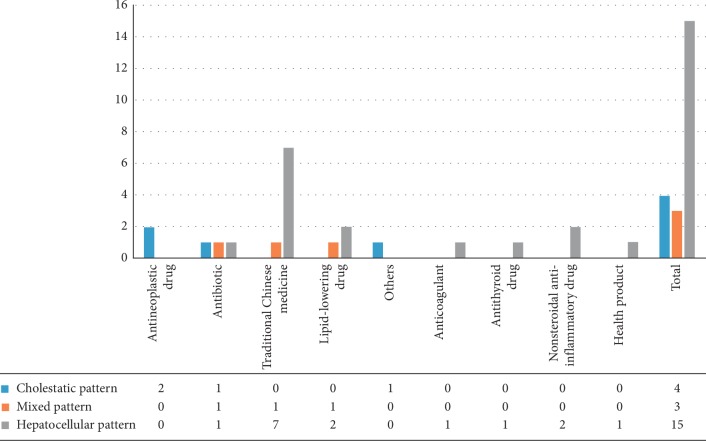
Patterns of liver injury in patients with simultaneous liver and kidney injuries.

There were 22 patients with simultaneous liver and kidney injuries with available ALT and ALP results. The following criteria for classifying the clinical pattern of drug-induced liver injury (DILI) were used [5, 6]: “*R* value = (ALT/ULN)/(ALP/ULN), hepatocellular pattern of DILI = *R* ≥ 5; mixed pattern of DILI = *R* > 2 and <5; and cholestatic pattern of DILI = *R* ≤ 2.” The results showed that, in 22 patients with simultaneous liver and kidney injuries, there were 15 (68.18%) with the hepatocellular pattern, 3 (13.64%) with the mixed pattern, and 4 (18.18%) with the cholestatic pattern. These results are shown in Figure 3. Therefore, we speculate that patients with a hepatocellular pattern of DILI are most likely to suffer from kidney injury, and traditional Chinese medicines are more likely to cause the hepatocellular pattern. It should be stated that the *R* value was able to be calculated in only 22 of 32 patients with simultaneous liver and kidney injuries, so the results of this study about the injury patterns and their association with renal injury may not be accurate.

We compared the levels of creatinine, uric acid, and urea nitrogen in patients with three patterns of liver injury. The *P* value for creatinine was 0.002 (<0.05). The difference was statistically significant. The average creatinine level in patients with the cholestatic pattern of liver injury was higher than that in the patients with the hepatocellular pattern and mixed pattern, as shown in Table 4.

### 3.3. Analysis of Acute Drug-Induced Liver and Kidney Injuries

(1) The patients with kidney injury were divided into 105 with isolated kidney injury and 32 with simultaneous liver and kidney injury group. We compared the levels of serum creatinine, uric acid, urea nitrogen, and urinary protein and microscopic hematuria between the two groups. The *P* value for urinary protein was 0.028 (<0.05). There was a significant difference. The results are shown in Table 5.
(2) The patients with liver injury were divided into 25 with the isolated liver injury group and 32 with the simultaneous liver and kidney injury group. We compared the levels of alanine aminotransferase, glutamyl transpeptidase, glutamic oxalate aminotransferase, alkaline phosphatase, and total bilirubin between the two groups. The results showed that there was no significant difference in liver biochemical tests between the two groups, as shown in Table 6.
(3) Univariate analysis of acute drug-induced liver injury and kidney injury.
    ① Statistical analysis of count datasex and underlying diseases are nongenetic factors affecting drug-induced liver and/or kidney injury. We compared the sex, coronary heart disease, diabetes, and hypertension data between the three groups: the kidney injury group, the liver injury group, and the simultaneous liver and kidney injury group. These data were statistically analyzed used the chi square test. The results showed that the sex, coronary heart disease, diabetes mellitus, and hypertension of the three groups of patients are not significantly different (all *P* > 0.05), as shown in Table 7.
    ② Statistical analysis of measurement data:we compared the age, weight, blood cholesterol, triglyceride, fasting blood glucose, white blood cell counts and INR of the kidney injury group, the liver injury group, and the simultaneous liver and kidney injury group. The results showed that the *P* value of the comparison of the percentage of neutrophils in the three groups was 0.049 (<0.05). The *P* values for the comparisons of age, weight, cholesterol, triglyceride, fasting blood sugar, white blood cell counts, and INR were all >0.05, as shown in Table 8.
(4) Multivariate logistic analysis of acute drug-induced liver and/or kidney injury
The risk factors for drug-induced liver and/or kidney injury include hereditary factors and nonhereditary factors, in addition to the characteristics of the drug itself, such as the dosage, course of treatment, and drug interactions among the combination of drugs administered. Hereditary factors include polymorphisms of drug metabolism-related genes and genetic susceptibility. Nonhereditary factors include sex, age, and underlying diseases. We collected data on sex, age, weight, and laboratory tests for analysis.
    ① Risk factors for liver injury in patients with drug-induced renal injury
    According to the criteria of whether liver function damage occurred, 137 patients with drug-induced kidney injury (including 105 patients with isolated kidney injury and 32 patients with liver and kidney injuries) were divided into two groups: the group with liver injury and the group without liver injury. Possible risk factors such as sex, age, weight, and cholesterol and triglyceride levels considered the independent variables (*X*), and liver injury was considered the response variable (*Y* = 0, *Y* = 1). In univariate analysis, the *P* value of the percentage of neutrophils was less than 0.05. Therefore, the percentage of neutrophils was also included in the regression model as an independent variable. It was divided into four groups according to interquartile ranges. Logistic stepwise regression was performed using the forward LR method. The entrance standard was 0.05, while the deletion standard was 0.10. *P* < 0.05 indicated a correlation.
    The logistic regression model was adjusted for sex, age, weight, cholesterol levels, triglyceride levels, and other factors. The results showed that patients with a higher percentage of neutrophils had a lower risk of liver injury, as shown in Table 9.
    ②Risk factors for drug-induced liver injury
    All 162 patients were divided into a liver injury group and a nonliver injury group according to whether liver injury occurred. Possible risk factors such as sex, age, weight, and cholesterol and triglyceride levels considered the independent variables (*X*), and liver injury was considered the response variable (*Y* = 0, *Y* = 1).
    The logistic regression model was adjusted for factors such as sex, age, weight, cholesterol levels, and triglyceride levels. The results showed that patients with a higher percentage of neutrophils had a lower risk of liver injury, as shown in Table 10.
    ③Risk factors for kidney injury in patients with drug-induced liver injury
    According to the criteria of whether kidney injury occurred, 57 patients with drug-induced liver injury (including 25 patients with isolated liver injury and 32 patients with simultaneous liver and kidney injuries) were divided into two groups. Possible risk factors such as sex, age, weight, and cholesterol and triglyceride levels considered the independent variables (*X*), and kidney injury was considered the response variable (*Y* = 0, *Y* = 1). The results showed that there was no significant difference in the independent variable *X*.
    ④Risk factors for drug-induced kidney injury
    All 162 patients were divided into two groups according to whether kidney injury occurred. Possible risk factors such as sex, age, weight, and cholesterol and triglyceride levels considered the independent variables (*X*), and kidney injury was considered the response variable (*Y* = 0, *Y* = 1). The logistic regression analysis results showed that there was no significant difference in the independent variable *X*.

## 4. Discussion

Drug-induced liver or kidney injury is a type of damage to the liver or kidney caused by drugs or metabolites via direct toxicity or the induction of an immune response when the drug is metabolized, decomposed, or excreted through the liver and kidney. Drug-induced liver and kidney injuries are key factors affecting the interruption of new drug research and drug withdrawal due to safety concerns [7]. Organ damage is also an important factor that leads to the interruption of patients' treatment, which makes clinical diagnosis and treatment difficult. In recent years, an increasing number of new drugs have been used in the clinic, but the abuse of nonstandard drugs, overdoses, and irrational combinations of drugs, such as antibiotics, traditional Chinese medicines, and health products, are still common. Under the influence of many factors, such as unreasonable drug use and the interactions among drugs, drug-induced liver and kidney injuries, as the most important adverse drug reactions, have attracted increasing attention from clinicians. The ability to diagnose these conditions is also continually improving [8, 9]. Drug-induced liver and kidney injuries are easily missed due to the lack of specific clinical manifestations and biochemical and pathological changes. Clinicians should pay attention to the standardization and rational use of drugs, be vigilant in the use of drugs with hepatic and renal toxicity and pay attention to monitoring.

The liver is the most important organ for drug catabolism in vivo. According to “Guidelines for the Diagnosis and Treatment of Drug-Induced Liver Injury” published in “Journal of Practical Hepatology” in 2017 by Pharmaceutical Hepatology Group of Chinese Medical Association, the incidence of drug-induced liver injury in China accounts for 20% of hospitalized patients with acute liver injury. Drug-induced liver injury is the main cause of acute liver failure in the United States and the most common reason for the FDA to enact regulatory measures to approve drugs in the United States [10, 11]. There are more than 1,000 drugs known to cause liver injury. The pathogenesis of drug-induced liver injury can be summarized as follows [12]: (1) direct toxicity of the drug or metabolite for the liver or (2) a specific heterogeneous response [13]: mediated by the immune response or the metabolic response, including oxidative stress, mitochondrial damage [14], and the adaptive immune response. These reactions are related to individual genetic susceptibility but not to the dose or course of treatment.

The kidney is the main organ for excreting drugs and various metabolic end products. It has abundant blood flow, active metabolism, and many types of active enzymes. It is susceptible to drug effects and is very vulnerable to drug-induced damage [15]. The main pathogenesis of drug-induced renal injury is as follows [16]: (1) the direct toxicity of drugs or metabolites for the kidney, which is related to the dosage and course of drug use, is most likely to occur in the renal tubules, because of the active metabolism and easy accumulation of drugs in the renal tubules; (2) effects on renal blood flow can occur because some drugs can cause kidney vasoconstriction to reduce renal blood flow and renal perfusion, leading to a decrease in the glomerular filtration rate; (3) obstruction leads to kidney damage because with the acidification of renal tubules and urine pH changes, some drug solubility decreases, causing precipitation and deposition in the renal tubular cavity, which results in obstructive lesions in the kidney; (4) metabolic disorders; (5) the immune response mediates oxidative stress [17], mitochondrial damage, and the adaptive immune response.

At present, most of the studies on drug-induced liver or kidney injury have separated the patients with liver or kidney injury and analyzed the groups separately. Our study analyzed the patients with drug-induced liver and kidney injuries, the differences in liver and kidney injuries caused by the same drug, the differences in liver and kidney injuries caused by different drugs, and the risk factors for liver and kidney injuries, all of which provided guidelines for rational clinical drug use. The results of our research included the following aspects: hepatocyte pattern, mixed pattern, and cholestasis pattern.

(1) Drug-induced liver injury can be divided into the hepatocyte pattern, mixed pattern, cholestasis pattern, and others. In this study, there were significant differences in serum creatinine levels among patients with the three types of liver injury. The average serum creatinine level of patients with the cholestasis pattern was the highest, which may indicate that their kidney injury was worse than that in the patients with the other two patterns. The results of this study are consistent with those reported in the literature [18]. The mechanism may be related to the inflammatory reaction induced by cholestasis. When cholestasis occurs, because of the obstruction of the intrahepatic and extrahepatic bile ducts, excretion disorders develop. Bile stasis leads to elevated levels of bile acid, bilirubin, and endotoxin in the blood, which leads to extensive inflammation in vivo. The kidney is one of the most affected target organs, and the inflammation and oxidative stress in the kidney damages the epithelial cells of the renal tubules.
Among the 22 patients with simultaneous liver and kidney injuries with ALT and ALP results from the same blood sample, 68.18% had the hepatocyte pattern. Therefore, we speculate that patients with the hepatocellular pattern of liver injury are the most likely to suffer from kidney injury, and traditional Chinese medicines are more likely to cause the hepatocellular pattern. However, the sample size of this study is small, and further cases should be collected to increase the power of the statistical analysis.
(2) In this study, there were significant differences in the urinary protein level between patients with isolated kidney injury and those with liver and kidney injuries, but there were no significant differences in serum creatinine, uric acid, and urea nitrogen levels.
(3) In this study, the risk factors for drug-induced liver injury, kidney injury, and liver and kidney injuries were analyzed by using logistic regression. Risk factors were not identified for drug-induced kidney injury, while the percentage of neutrophils was an influencing factor for drug-induced liver injury.
In patients with drug-induced organ injury, patients with a higher percentage of neutrophils had a lower risk of liver injury. Saijou et al. [19] and Wang and Ding [20] found that some sRNAs in neutrophils has a protective effect against acute liver injury. The innate immune system in the human liver mainly consists of Kupffer cells (KCs), neutrophils, monocytes, and natural killer cells/natural killer T cells (NK/NKT cells) [21–23]. Neutrophils can aggregate into the hepatic microvascular system under the action of interleukin and play an important role in resisting infection, tissue damage, and inflammatory signals. They can also regulate immunity by inhibiting T cell proliferation and regulating B lymphocytes.
(4) In this study, the most common drugs causing liver and/or kidney injury were antineoplastic drugs, antibiotics, traditional Chinese medicines, lipid-lowering drugs, nonsteroidal anti-inflammatory drugs, antituberculosis drugs [24], antithyroid drugs, hormones, antiviral drugs, and sedative and psychotropic drugs. Antineoplastic drugs [25], antibiotics, and nonsteroidal anti-inflammatory drugs are more likely to lead to kidney injury, while traditional Chinese medicines and lipid-lowering drugs are more likely to lead to liver injury.
  1. In China, traditional Chinese medicines, antituberculosis drugs, antibiotics, and nonsteroidal anti-inflammatory drugs are common drugs that cause drug-induced liver injury, while *Tripterygium wilfordii*, Radix Notoginseng, and Radix Polygoni Multiflori rank highest in terms of hepatotoxicity among traditional Chinese medicines. In this study, the most common drugs causing acute liver injury were traditional Chinese medicines, lipid-lowering drugs, and antibiotics, which are in line with the general conditions in China.
  2. In China, common drugs that can cause kidney injury are antibiotics, contrast agents, nonsteroidal anti-inflammatory drugs, and anticancer drugs. Among antibiotics, aminoglycosides and *β*-lactams are the most common causes of kidney injury. China is a country with a high incidence of tuberculosis; therefore, the incidence of kidney injury caused by antituberculosis drugs such as rifampicin and acyclovir has been increasing. In this study, the most common drugs causing acute kidney injury were antineoplastic drugs, antibiotics, and traditional Chinese medicines.

There are limitations in our study. (1) This is a retrospective analysis lacking some detailed data including detection of ALP and urinary sediment and medical history of the sequence of drug-induced liver and/or kidney injury. (2) In the research subjects, there were 32 patients suffering from simultaneous liver and kidney injuries, among which there were 22 (68.75%) patients whose *R* value could be calculated as these people had detected ALT and ALP simultaneously in the blood sample. So the conclusions drawn about the injury patterns and their association with renal injury may be inaccurate. (3) Although our study selected all patients met the inclusion criteria in our hospital from 2008 to 2018, the number of patients with kidney injury is relatively large, while those with liver injury is small. So there may be biases in the analysis results.

The liver and kidney, as the main organs involved in drug metabolism and excretion in vivo, are inextricably linked [26]. However, there have been few studies conducting a joint analysis of drug-induced liver injury and kidney injuries. In this study, we studied the liver and kidney injuries caused by different drugs and the risk factors for drug-induced liver and/or kidney injury to provide guidance for clinical practice. Because the research subjects were obtained from the same hospital and the sample size was limited, further study should be performed, with an increased sample size and reduced sampling bias, and prospectively if possible, to further elucidate the relationship between drug-induced liver injury and kidney injury.

## Figures and Tables

**Table 1:** Comparison of liver and renal biochemical tests between antibiotic group and antineoplastic group.

	Antibiotic group	Antineoplastic drug group	Statistic	*P* value
Alanine aminotransferase (U/L)	*M* = 57.2, *Q* = 141.7	68.769 ± 48.5607	892.5	0.659
Total bilirubin (*μ*mol/L)	*M* = 10.53, *Q* = 19.09	13.8509 ± 8.3966	914.5	0.691
Blood urea nitrogen (mmol/L)	8.55 ± 7.116	*M* = 4.97, *Q* = 3.12	843.5	0.234
Uric acid (*μ*mol/L)	304.95 ± 196.721	293.0707 ± 136.5688	0.305	0.761
Creatinine (*μ*mol/L)	118.463 ± 83.8618	*M* = 66.91, *Q* = 37.11	800	0.123

*M* means median, and *Q* means interquartile range.

**Table 2:** Differences in renal injury in patients with isolated renal injury and simultaneous liver and kidney injuries induced by antibiotics.

Antibiotic	Isolated renal injury group	Simultaneous liver and kidney injury group	Statistics of *T* test	*P* value
Creatinine (*μ*mol/L)	128.6122 ± 88.5228	103.8233 ± 72.72	0.644	0.524
Uric acid (*μ*mol/L)	279.943 ± 167.493	416.65 ± 298.5011	−1.454	0.159
Urea nitrogen (mmol/L)	8.4944 ± 7.8330	3.6633 ± 1.7097	1.35	0.186

	Isolated renal injury group	Simultaneous liver and kidney injury group	Fisher's exact test	*P* value
Urine protein				
Yes	5	1		0.73
No	14	3		
Microscopic hematuria				
Yes	8	2		0.596
No	11	2		

**Table 3:** Differences in liver biochemical tests in patients with isolated liver injury and liver and kidney injuries induced by antibiotics.

Antibiotic	Isolated liver injury group	Simultaneous liver and kidney injury group	Statistics of *T* test or Mann–Whitney *U* test	*P* value
Alanine aminotransferase (U/L)	463.6667 ± 114.7687	507.8667 ± 277.9499	−0.257	0.804
Glutamyl transpeptidase (U/L)	254.3 ± 281.086	606.7333 ± 126.2854	−1.981	0.119
Alkaline phosphatase (U/L)	203.1333 ± 98.8588	300.7333 ± 86.1836	−1.289	0.267
Total bilirubin (*μ*mol/L)	58.4967 ± 78.5981	1.2499 ± 1.06243	1.261	0.276

INR: international normalized ratio of prothrombin.

**Table 4:** Comparison of renal injury in patients with three patterns of liver injury.

Patterns of liver injury	Cholestatis pattern	Mixed pattern	Hepatocellular pattern	Statistics of Kruskal–Wallis test	*P* value
Creatinine (*μ*mol/L)	156.425 ± 63.4374	81.5333 ± 7.6173	62.3027 ± 14.1525	12.638	0.002
Uric acid (*μ*mol/L)	581.45 ± 405.9916	326.5667 ± 92.3952	272.9462 ± 93.5693	2.945	0.229
Urea nitrogen (mmol/L)	7.2375 ± 4.2323	4.2667 ± 0.7506	4.4973 ± 1.0801	2.819	0.244

**Table 5:** Comparison of renal function indicators between the isolated kidney injury group and the simultaneous liver and kidney injury group.

	Isolated kidney injury group	Simultaneous liver and kidney injury group	Statistics of Mann–Whitney *U* test	*P* value

Creatinine (*μ*mol/L)	*M* = 75.28, *Q* = 63.85	*M* = 70.47, *Q* = 26.23	1535.5	0.462
Uric acid (*μ*mol/L)	296.6806 ± 129.8390	*M* = 307.25, *Q* = 137.93	906.5	0.371
Urea nitrogen (mmol/L)	*M* = 5.29, *Q* = 4.44	*M* = 4.885, *Q* = 1.665	1341.5	0.085

	Isolated kidney injury group	Simultaneous liver and kidney injury group	Statistics of chi square	*P* value

Urine protein				
Yes	20	9	4.803	0.028
No	52	7		
Microscopic hematuria				
Yes	25	3	1.539	0.215
No	47	13		

*M* means median, and *Q* means interquartile range.

**Table 6:** Comparison of liver biochemical tests between the isolated liver injury group and the simultaneous liver and kidney injury group.

	Isolated liver injury group	Simultaneous liver and kidney injury group	Statistics of *T* test or Mann–Whitney *U* test	*P* value
Alanine aminotransferase (U/L)	600.79 ± 387.382	671.22 ± 676.185	−0.464	0.644
Glutamyl transpeptidase (U/L)	*M* = 129.5, *Q* = 245	436.7636 ± 607.1882	157	0.174
Glutamic oxaloacetic transaminase (U/L)	468.57 ± 330.24	419.5582 ± 352.5932	0.388	0.701
Alkaline phosphatase (U/L)	197.0063 ± 133.1402	222.0682 ± 123.7677	−0.597	0.554
Total bilirubin (*μ*mol/L)	75.6354 ± 108.2917	58.6334 ± 58.4483	0.755	0.454

*M* means median, and *Q* means interquartile range.

**Table 7:** Univariate analysis of count data of kidney injury group, simultaneous liver and kidney injury group, and liver injury group.

	Kidney injury group	Liver and kidney injury group	Liver injury group	Statistics of chi square	*P* value
*Sex*					
Male	43	16	9	1.258	0.533
Female	62	16	16		
*Coronary heart disease*					
Yes	10	2	2	0.348	0.84
No	95	30	23		
*Diabetes*					
Yes	12	3	2	0.306	0.858
No	93	29	23		
*Hypertension*					
Yes	21	8	4	0.726	0.696
No	84	24	21		

**Table 8:** Univariate analysis of measurement data of kidney injury group, simultaneous liver and kidney injury group, and liver injury group.

	Kidney injury group	Liver and kidney injury group	Liver injury group	*F* value of variance analysis	*P* value

Age (y)	51.23 ± 16.911	50.88 ± 14.318	49.76 ± 14.967	0.084	0.92
Weight (kg)	63.25 ± 11.274	65.75 ± 9.247	64.78 ± 11.698	0.71	0.493
Cholesterol (mmol/L)	4.3516 ± 1.6511	4.2231 ± 1.1637	3.8328 ± 0.9036	1.257	0.287
Percentage of neutrophils (%)	67.1323 ± 19.1865	57.9312 ± 16.0345	59.2480 ± 32.5661	3.073	0.049

	Kidney injury group	Liver and kidney injury group	Liver injury group	Statistics of rank-sum test	*P* value

Triglyceride (mmol/L)	*M* = 1.37, *Q* = 0.975	1.6725 ± 0.8626	1.5056 ± 0.6379	2.454	0.293
Fasting blood glucose (mmol/L)	*M* = 5.4150, *Q* = 1.4450	5.6264 ± 1.4650	5.9216 ± 2.0646	0.962	0.618
White blood cell (10^9^/L)	6.9263 ± 4.8850	*M* = 5.73, *Q* = 2.995	5.9713 ± 2.0841	1.062	0.588
INR	*M* = 0.99, *Q* = 0.125	1.099 ± 0.1828	1.0336 ± 0.1994	3.298	0.192

INR: the international standardized ratio. *M* means median, and *Q* means interquartile range.

**Table 9:** Regression analysis results for liver injury in patients with drug-induced renal injury.

Percentage of neutrophils	*P* value	OR (95% CI)	Mean of no liver injury group	Mean of liver injury group
<53.15	0.054	1	67.1323	57.9312
53.15–68.2	0.302	0.582 (0.208–1.627)		
68.2–76.8	0.115	0.419 (0.142–1.235)		
>76.8	0.008	0.156 (0.040–0.617)		
*P* for trend	0.005	0.574 (0.390–0.846)		

OR: odds ratio.

**Table 10:** Regression analysis results for drug-induced liver injury.

Percentage of neutrophils	*P* value	OR (95% CI)	Mean of no liver injury group	Mean of liver injury group
<50.6	0.003	1	67.1323	58.5088
50.6–67.3	0.187	0.554 (0.231–1.330)		
67.3–76.25	0.007	0.270 (0.105–0.696)		
>76.25	0.001	0.171 (0.061–0.477)		
*P* for trend	0.000	0.545 (0.396–0.749)		

OR: odds ratio.

## Data Availability

The clinical data used to support the findings of this study are included within the supplementary information file.

## Abbreviations

ADR: Adverse drug reactions
AKI: Acute kidney injury
ALP: Alkaline phosphatase
ALT: Alanine aminotransferase
AST: Aspartate aminotransferase
CI: Confidence interval
eGFR: Estimated glomerular filtration rate
HRD: Hepatorenal disorder
INR: International normalized ratio
KCs: Kupffer cells
K/DOQI: Kidney disease outcomes quality initiative
K-S: Kolmogorov–Smirnov
LR: Likelihood ratio
MELD: Model for end-stage liver disease
MDRD: Modification of diet in renal diseases
NK/NKT cells: Natural killer cells/natural killer T cells
OR: Odds ratio
scr: Serum creatinine
SPSS: Statistical product and service solutions
TB: Total bilirubin
*T* test: Student's *t* test
*U* test: Mann–Whitney *U* test.

## References

[1] van Montfoort J., Hagenbuch B., Groothuis G., Koepsell H., Meier P., Meijer D. (2003). Drug uptake systems in liver and kidney. *Current Drug Metabolism*.

[2] Aithal G. P., Watkins P. B., Andrade R. J. (2011). Case definition and phenotype standardization in drug-induced liver injury. *Clinical Pharmacology & Therapeutics*.

[3] Andrade R. J., Chalasani N., Bjornsson E. S. (2019). Drug-induced liver injury. *Nature Reviews Disease Primers*.

[4] Usui J., Yamagata K., Imai E. (2016). Clinical practice guideline for drug-induced kidney injury in Japan 2016: digest version. *Clinical and Experimental Nephrology*.

[5] Bénichou C. (1990). Criteria of drug-induced liver disorders: report of an international consensus meeting. *Journal of Hepatology*.

[6] Danan G., Benichou C. (1993). Causality assessment of adverse reactions to drugs-I: a novel method based on the conclusions of international consensus meetings: application to drug-induced liver injuries. *Journal of Clinical Epidemiology*.

[7] Hayashi P. (2016). Drug-induced liver injury network causality assessment: criteria and experience in the United States. *International Journal of Molecular Sciences*.

[8] Teschke R., Danan G. (2018). Molecular research on drug induced liver injury. *International Journal of Molecular Sciences*.

[9] Weber E. J., Lidberg K. A., Wang L. (2018). Human kidney on a chip assessment of polymyxin antibiotic nephrotoxicity. *JCI Insight*.

[10] Fontana R. J., Watkins P. B., Bonkovsky H. L. (2009). Drug-induced liver injury network (DILIN) prospective study. *Drug Safety*.

[11] Liu Z., Shi Q., Ding D., Kelly R., Fang H., Tong W. (2011). Translating clinical findings into knowledge in drug safety evaluation—drug induced liver injury prediction system (DILIps). *PLoS Computational Biology*.

[12] Ye H., Nelson L. J., Moral M. G. D., Martínez-Naves E., Cubero F. J. (2018). Dissecting the molecular pathophysiology of drug-induced liver injury. *World Journal of Gastroenterology*.

[13] Radovanovic M., Dushenkovska T., Cvorovic I. (2018). Idiosyncratic drug-induced liver injury due to ciprofloxacin: a report of two cases and review of the literature. *American Journal of Case Reports*.

[14] Aleo M. D., Luo Y., Swiss R., Bonin P. D., Potter D. M., Will Y. (2014). Human drug-induced liver injury severity is highly associated with dual inhibition of liver mitochondrial function and bile salt export pump. *Hepatology*.

[15] Fuchs T. C., Hewitt P. (2011). Biomarkers for drug-induced renal damage and nephrotoxicity-an overview for applied toxicology. *The AAPS Journal*.

[16] Awdishu L., Mehta R. L. (2017). The 6R’s of drug induced nephrotoxicity. *BMC Nephrology*.

[17] Hosohata K. (2016). Role of oxidative stress in drug-induced kidney injury. *International Journal of Molecular Sciences*.

[18] Krones E., Wagner M., Eller K., Rosenkranz A. R., Trauner M., Fickert P. (2015). Bile acid-induced cholemic nephropathy. *Digestive Diseases*.

[19] Saijou E., Enomoto Y., Matsuda M. (2018). Neutrophils alleviate fibrosis in the CCl_4_ -induced mouse chronic liver injury model. *Hepatology Communications*.

[20] Wang S., Ding W.-X. (2017). A small RNA in neutrophils protects against acute-on-chronic alcoholic liver injury. *Gut*.

[21] Tujios S., Fontana R. J. (2011). Mechanisms of drug-induced liver injury: from bedside to bench. *Nature Reviews Gastroenterology & Hepatology*.

[22] Ju C., Reilly T. (2012). Role of immune reactions in drug-induced liver injury (DILI). *Drug Metabolism Reviews*.

[23] Amulic B., Cazalet C., Hayes G. L., Metzler K. D., Zychlinsky A. (2012). Neutrophil function: from mechanisms to disease. *Annual Review of Immunology*.

[24] Bao Y., Ma X., Rasmussen T. P., Zhong X.-B. (2018). Genetic variations associated with anti-tuberculosis drug-induced liver injury. *Current Pharmacology Reports*.

[25] Izzedine H., Perazella M. A. (2017). Anticancer drug-induced acute kidney injury. *Kidney International Reports*.

[26] Nawaratne S., Brien J. A. E., Seeman E. (1998). Relationships among liver and kidney volumes, lean body mass and drug clearance. *British Journal of Clinical Pharmacology*.

